# More Than Just Mosquito Bites: A Travel-Associated Case of Cutaneous Small-Vessel Vasculitis

**DOI:** 10.7759/cureus.90677

**Published:** 2025-08-21

**Authors:** Allan Zhou, Shao Hui Koh

**Affiliations:** 1 Emergency Medicine, Sengkang General Hospital, Singapore, SGP

**Keywords:** cutaneous small-vessel vasculitis, leukocytoclastic vasculitis, palpable purpura, post-infectious vasculitis, travel-related rash

## Abstract

A 39-year-old man presented with a bilateral lower limb pruritic and purpuric rash following travel to Chongqing, China. He was initially treated for cellulitis abroad. On arrival, his rash was noted, and the initial workup revealed raised inflammatory markers with a CRP of 252 mg/L and an ESR of 80 mm/hr. Examination of the lower limb reveals symmetrical, non-blanchable purpuric plaques with post-inflammatory hyperpigmentation. He was admitted for further inpatient treatment. Serological workup for autoimmune vasculitis was negative. Urine phase contrast microscopy showed 12% dysmorphic red blood cells, below the threshold typically seen in glomerular bleeding. A punch biopsy revealed minimal, superficial perivascular lymphocytic infiltrate without classic vasculitic features, likely due to timing post-acute inflammation. The clinical workup and examination findings support a diagnosis of post-infectious cutaneous small-vessel vasculitis (CSVV).

This case demonstrates the classic distribution and morphology of CSVV. Early recognition of CSVV facilitates prompt systemic evaluation and discontinuation of potential triggers and avoids unnecessary antibiotic use.

## Introduction

Cutaneous small-vessel vasculitis (CSVV) is a form of vasculitis confined to the skin, presenting with clusters of palpable purpura on the lower limbs [1]. It is characterized by inflammation and damage to small blood vessels in the skin, typically caused by neutrophilic infiltration from immune complex deposition. In the 2012 Revised Chapel Hill Consensus Nomenclature, CSVV is classified as a single-organ cutaneous vasculitis, whereas systemic vasculitis may present with similar cutaneous findings but involve other organs, most commonly involving the renal, musculoskeletal, and gastrointestinal systems [2,3].

CSVV is frequently associated with an underlying trigger or systemic condition, with about half of all cases attributed to identifiable causes and the remainder classified as idiopathic [3]. Possible triggers include recent infections, medications, vaccinations, autoimmune diseases, and malignancy [2].

The prognosis of CSVV is generally favorable, with most patients experiencing self-resolution without relapse. In contrast, systemic vasculitis carries a higher risk of relapse, greater treatment burden, and worse survival outcomes [3].

It is therefore important that patients presenting with cutaneous vasculitis undergo evaluation for possible systemic triggers and exclude the possibility of underlying systemic vasculitis.

## Case presentation

A 39-year-old man with a history of hyperlipidemia presented to our emergency department with an evolving rash over his lower limbs and fever following recent travel to Chongqing, China. He first noted small, red, discrete pruritic papules over his legs and arms while on a river cruise (day one of illness), which he attributed to insect bites. Over the subsequent four days, he developed a tender, erythematous swelling over the right medial ankle, with fever developing on day five of illness. He was admitted to a hospital in China on day five and treated for cellulitis with a one-day course of intravenous cefuroxime and discharged with oral amoxicillin-clavulanate. This initial tender erythematous swelling over the right medial ankle improved over the next few days. However, the initial red pruritic papules over his legs started developing into larger purpuric plaques over both his lower limbs, accompanied by a fever that had persisted for five days. On return from his trip on day nine of illness, he presented to our hospital. He did not have bleeding manifestations, eye redness, mouth ulcers, abdominal pain, bodily swelling, arthralgia, hematuria, or respiratory or neurological symptoms. He also did not have any prior skin conditions, drug allergies, or personal or family history of autoimmune conditions or coagulopathy.

**Figure 1:**
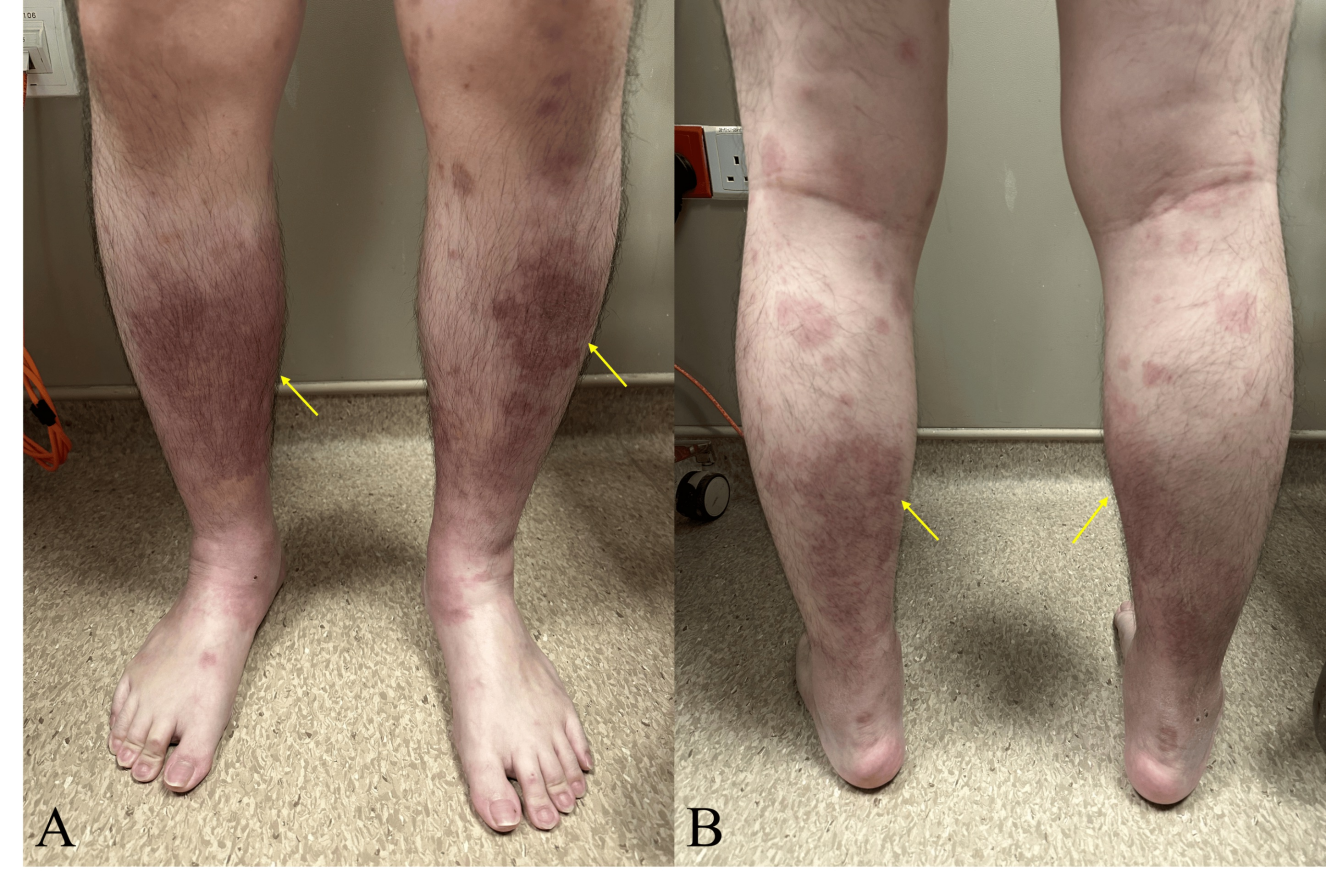
Images of bilateral lower limbs Anterior (A) and posterior (B) views of bilateral lower limbs with palpable, non-blanchable, purpuric plaques clustered over the shins. Photographs were taken on day nine of illness.

On examination, bilateral shins exhibited tender, non-blanchable purpuric plaques as seen in Figure 1, while the forearms showed scattered hyperpigmented macules. There was no bodily edema, eye or oral mucosal changes, synovitis, abdominal tenderness, or skin changes seen elsewhere. Examination of his heart and lungs was also unremarkable. His vital signs were within normal ranges with a blood pressure of 123/80, a heart rate of 94, a temperature of 36.8°C, and saturation on room air.

Initial investigations showed raised inflammatory markers and transaminase levels. Full blood count and renal function were otherwise normal; urinalysis revealed microscopic hematuria. Laboratory test results and corresponding reference ranges are summarized in Table 1. The chest radiograph performed was normal.

**Table 1:** Investigation results on admission

Test	Result	Reference Range
C-reactive Protein (CRP)	252 mg/L	<5 mg/L
Erythrocyte Sedimentation Rate (ESR)	80 mm/hr	<20 mm/hr
White Blood Cells	8.91 ×10⁹/L	4.0–10.0 ×10⁹/L
Hemoglobin	13.6 g/dL	13.5–17.5 g/dL
Platelets	273 ×10⁹/L	150–400 ×10⁹/L
Alanine Aminotransferase (ALT)	232 U/L	7–56 U/L
Aspartate Aminotransferase (AST)	181 U/L	10–40 U/L
Urinalysis – Red Blood Cells	324 /µL	<5 /µL

He was diagnosed with vasculitis of unclear etiology and was admitted for further evaluation.

Serological workup for autoimmune and infective vasculitides was unremarkable. Antinuclear antibody, antineutrophil cytoplasmic antibody, anti-double-stranded DNA, anti-myeloperoxidase antibody, and anti-proteinase-3 antibody were negative. Serum immunoglobulin A and complement components C3 and C4 were within normal limits, making an autoimmune etiology unlikely. Viral serologies were negative for hepatitis B, C, and human immunodeficiency virus. Urine phase contrast microscopy revealed 12% dysmorphic red blood cells, below the threshold typically seen in glomerular bleeding of >40% [4]. Although microscopic hematuria and transaminitis were present, the urine findings were not accompanied by proteinuria or impaired renal function. The hepatic enzyme elevations were modest and not associated with jaundice or other systemic features. Taken together, these findings did not indicate clinically significant renal or hepatic involvement suggestive of systemic vasculitis. Respiratory multiplex PCR was positive for coronavirus HKU1 and human rhinovirus/enterovirus.

A punch biopsy was performed on day 10 of illness, and histology revealed minimal, superficial perivascular lymphocytic infiltrate without fibrinoid necrosis, leukocytoclasia, or erythrocyte extravasation. Immunofluorescence was negative for immune complex deposition (C3, IgA, IgG, IgM, and fibrin). The absence of classic vasculitis features may be attributed to the timing of the biopsy, which was performed after the acute inflammatory phase. Although the respiratory multiplex was positive for coronavirus HKU1 and human rhinovirus/enterovirus, it is not possible to establish a definitive causal trigger. When interpreted in conjunction with the clinical picture, these findings support the diagnosis of post-infectious CSVV. 

The patient’s rash improved with continued oral cefuroxime and doxycycline and application of topical clobetasol propionate and was resolving as of discharge, approximately two weeks from the onset of initial pruritic papules and around five days after the evolution into purpuric plaques. While the improvement of the rash occurred in parallel with oral antibiotics, the clinical course was more consistent with self-limited CSVV. To our knowledge, there has been no recurrence of his rash.

## Discussion

Initial recognition of CSVV is clinical and based on the morphology and distribution of palpable purpura. Bilateral cellulitis and coagulopathies should be considered and excluded based on history and investigation. The vasculitis may be skin-limited or affect multiple bodily systems. Features in the history suggestive of the latter include fever, abdominal pain, bodily swelling, arthralgia, hematuria, and/or neurologic symptoms.

First-line investigations include a full blood count, renal and liver function tests, coagulation profile, C-reactive protein (CRP), erythrocyte sedimentation rate (ESR), and urinalysis. A chest radiograph should also be performed if the patient has respiratory symptoms.

Definitive diagnosis of CSVV requires confirmation via skin biopsy. Histologically, it is characterized by neutrophilic infiltration with leukocytoclasia and fibrinoid necrosis of postcapillary venules. The presence of these features on hematoxylin and eosin (H&E) staining, ideally from a lesion less than 48 hours old, provides high diagnostic yield [1]. Direct immunofluorescence can aid in classification by identifying immune complex deposition patterns, which have diagnostic and prognostic value [5].

In patients with systemic vasculitis, serologic testing for hepatitis B and C, antinuclear antibodies, anti-neutrophil cytoplasmic antibodies, serum IgA, and complement levels should be performed to elicit the underlying causes [6].

Management of CSVV is largely supportive. Most cases are self-limiting and self-resolve within weeks after removal of the inciting trigger. In the absence of systemic disease, the prognosis is good, with complete resolution of the rash in most patients [7]. Recurrence rates are reported to be between 10% and 25%, especially in cases associated with autoimmune conditions or extensive cutaneous involvement [3].

## Conclusions

This case highlights the classic distribution and morphology of CSVV. Although histological results did not demonstrate classic histopathological features, likely due to the timing of the biopsy, the overall picture was consistent with CSVV. Emergency physicians need to maintain a high index of suspicion for CSVV in returning travelers or patients with unexplained purpura. Early recognition of CSVV allows for prompt systemic workup, discontinuation of potential triggers, and limits unnecessary antibiotic use.
